# Nutritional Status of Under-five Children Living in an Informal Urban Settlement in Nairobi, Kenya

**DOI:** 10.3329/jhpn.v29i4.8451

**Published:** 2011-08

**Authors:** Beatrice Olack, Heather Burke, Leonard Cosmas, Sapna Bamrah, Kathleen Dooling, Daniel R. Feikin, Leisel E. Talley, Robert F. Breiman

**Affiliations:** ^1^Kenya Medical Research Institute, Nairobi, Kenya; ^2^International Emerging Infections Program, Centers for Disease Control and Prevention, Kenya,; ^3^Centers for Disease Control and Prevention, Atlanta, GA, USA,; ^4^Peel Public Health Unit, Brampton, Canada

**Keywords:** Child nutrition, Child nutrition disorders, Child nutritional status, Cross-sectional studies, Food security, Informal settlement, Slums, Kenya

## Abstract

Malnutrition in sub-Saharan Africa contributes to high rates of childhood morbidity and mortality. However, little information on the nutritional status of children is available from informal settlements. During the period of post-election violence in Kenya during December 2007–March 2008, food shortages were widespread within informal settlements in Nairobi. To investigate whether food insecurity due to post-election violence resulted in high prevalence of acute and chronic malnutrition in children, a nutritional survey was undertaken among children aged 6-59 months within two villages in Kibera, where the Kenya Medical Research Institute/Centers for Disease Control and Prevention conducts population-based surveillance for infectious disease syndromes. During 25 March–4 April 2008, a structured questionnaire was administered to caregivers of 1,310 children identified through surveillance system databases to obtain information on household demographics, food availability, and child-feeding practices. Anthropometric measurements were recorded on all participating children. Indices were reported in z-scores and compared with the World Health Organization (WHO) 2005 reference population to determine the nutritional status of children. Data were analyzed using the Anthro software of WHO and the SAS. Stunting was found in 47.0% of the children; 11.8% were underweight, and 2.6% were wasted. Severe stunting was found in 23.4% of the children; severe underweight in 3.1%, and severe wasting in 0.6%. Children aged 36-47 months had the highest prevalence (58.0%) of stunting while the highest prevalence (4.1%) of wasting was in children aged 6-11 months. Boys were more stunted than girls (p<0.01), and older children were significantly (p<0.0001) stunted compared to younger children. In the third year of life, girls were more likely than boys to be wasted (p<0.01). The high prevalence of chronic malnutrition suggests that stunting is a sustained problem within this urban informal settlement, not specifically resulting from the relatively brief political crisis. The predominance of stunting in older children indicates failure in growth and development during the first two years of life. Food programmes in Kenya have traditionally focused on rural areas and refugee camps. The findings of the study suggest that tackling childhood stunting is a high priority, and there should be fostered efforts to ensure that malnutrition-prevention strategies include the urban poor.

## INTRODUCTION

Malnutrition is a serious medical condition marked by a deficiency of energy, essential proteins, fats, vitamins, and minerals in a diet. Over 10 million children aged less than five years (under-five children) die annually from preventable and treatable illnesses — almost all these deaths occur in poor countries (1). Malnutrition contributes to more than one-third of all deaths of under-five children (2). Currently, 195 million under-five children are affected by malnutrition 90% of them live in sub-Saharan Africa and South Asia. At least 20 million children suffer from severe acute malnutrition (SAM), and another 175 million are undernourished (3). Malnutrition is the most recognizable and perhaps most untoward consequence of poverty in children (4).

In sub-Saharan Africa and in most developing countries, extreme urban poverty is concentrated in temporary or informal squatter settlements and slum areas. Infrastructure is not keeping up with massive urbanization throughout Africa. As a result, each year a growing number of people live within informal settlements. Informal settlements are made up of improvised dwellings often made from scrap materials, such as corrugated metal sheets, plywood, and polythene-sheets. They tend to be densely populated and characterized by limited basic services and infrastructure for providing clean water, sanitation facilities, solid-waste management, roads, drainage, and electricity, if any is available at all. In concert with poverty, a number of factors within informal settlements, including overcrowding, substandard housing, unclean and insufficient quantities of water, and inadequate sanitation, contribute to a high incidence of infectious diseases and to significant rates of childhood mortality (5).

It is estimated that more than 60% of Nairobi residents live in informal settlements (6) where poverty, combined with population density and poor sanitation, is readily evident. The 2008 Kenya Demographic and Health Survey showed that 35.3% of under-five children were stunted nationwide, 6.7% were wasted, and 16.3% were underweight (7). The report suggested that the greatest burden of malnutrition was in rural areas.

Kenya experienced a period of civil unrest following the December 2007 general election. The Kibera informal settlement, the largest contiguous slum in Africa, was a focal point for substantial post-election violence. The associated chaos disrupted sources of livelihoods, reducing access of Kibera residents to food and basic services. The emergence of inter-tribal hostilities in Kibera forced some residents to move to camps of internally-displaced persons (IDPs) where they remained for several months. During the period of civil unrest, food shortages and rising food prices were dramatic. News reports from health facilities suggested that moderately-malnourished children were becoming severely malnourished due to acute food shortages (8). It was anticipated that rates of malnutrition would continue to rise due to insecurity and lack of access to food. In response to concerns about inadequate nutrition for children during the post-election violence, we conducted an assessment to determine the nutritional status of children aged 6-59 months in two villages in Kibera where the Kenya Medical Research Institute (KEMRI) and Centers for Disease Control and Prevention (CDC) are collaborating on population-based surveillance for major infectious disease syndromes and for emerging and re-emerging pathogens. We compared data obtained during this survey with data from a nutritional survey conducted in 2005 in the same villages before the start of the KEMRI/CDC surveillance activities. (Dooling K *et al*. Unpublished observations).

## MATERIALS AND METHODS

This cross-sectional study was conducted in Kibera during 25 March–4 April 2008. The study was conducted in two villages (Gatwikira and Soweto)where KEMRI/CDC has conducted population-based surveillance for infectious disease syndromes since 2005 (9) which, during the time of the nutritional study, included biweekly household visits to detect acute diseases, such as pneumonia, diarrhoeal diseases, and febrile illnesses (10).

A week-long intensive training (including anthropometric measurements) was held for field workers to prepare for the nutritional survey. Children aged 6-59 months are considered to be particularly susceptible to acute nutritional stress; thus, surveying this age-group provided an indication of the severity of undernutrition among all people living in a geographic area under similar conditions. Using the KEMRI/CDC population-based surveillance database, we selected all households within the surveillance area with at least one child aged 6-59 months for inclusion in the survey. Trained field workers then visited all the selected households. If no one was in the home during the initial visit, two repeat visits were made to all eligible households. The survey covered all children aged 6-59 months currently present in the surveillance area. Due to the post-election violence, approximately two-thirds of the residents had left Kibera and had not yet returned; so, they could not be surveyed. In addition, of 1,476 children present in the surveillance area, 1,310 (89%) were available at the time of visit to the home during the survey. A structured questionnaire was administered to caregivers of children to obtain information on household demographics, socioeconomic factors, availability of foods, and child-feeding practices. To assess whether malnutrition was linked to morbidi-ty, we analyzed corresponding data (for each surveyed child) collected during home-visits for the disease surveillance.

Anthropometric measurements were taken for all children, aged 6-59 months, included in the cross-sectional survey to assess their nutritional status. Length/Height Board of the United Nations Children's Funds was used by the field workers after undergoing training to measure the height or length of children. Readings of heights were taken to the nearest centimetre. Portable (Seca Model 881) scales were used for measuring weight of children dressed in light clothing. The scales were checked for accuracy and calibrated every morning using standard known weights. Weights were recorded to the nearest 0.1 kg. Children who could not stand on the scale were weighed with the respondent, then the respondent was weighed alone, and the difference was used for obtaining weight of the child.

### Analysis of data

Anthropometric indices were calculated using reference medians recommended by the World Health Organization (WHO) and classified according to standard deviation units (z-scores), based on the WHO criteria (11). Wasting (weight-for-height z-score–WHZ) indicates thinness. It is usually the result of recent nutritional deficiency and is affected by seasonal shifts associated with availability of foods and/or prevalence of disease. A WHZ of <-2 defines the presence of acute malnutrition (wasting). Stunting, represented by low height-for-age z-score (HAZ), results from extended periods of inadequate food intake, poor dietary quality, increased morbidity, or a combination of these factors. A HAZ of <-2 defines chronic malnutrition (stunting). Weight-for-age z-score (WAZ) is essentially a composite of weight-for-height and height-for-age, thus a measure of both acute and chronic malnutrition. A WAZ of <-2 is used for defining a child as underweight. A z-score of <-3 defines severe levels of each of the indices. The Anthro software of WHO was used for analyzing the nutritional status of children, and all other analyses were done using the SAS (version 9.1) (12). The chi-square test was used for assessing the significance of nutritional indices and various independent variables of interest. To measure the impact of food insecurity on children, the results from this survey were compared with the results of a similar nutritional survey conducted in the same area in Kibera in 2005.

### Ethical approval

The head of household gave informed consent for home-visits. The protocol and consent forms were reviewed and approved by the Ethical Review Boards of the KEMRI (no. 932) and the Institutional Review Board of the CDC (no. 4566).

## RESULTS

**Fig. 1.**
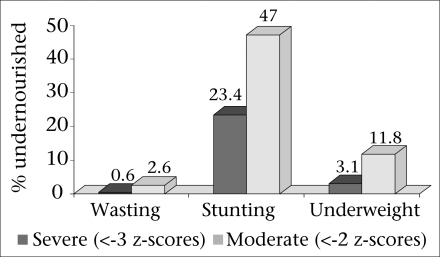
Prevalence of severe and moderate malnutrition

Anthropometric measurements were available for 1,245 (592 males and 653 females) of the 1,310 children surveyed. Stunting was found in 47% [95% confidence interval (CI) 44.2-49.8] of the children while 11.8% (95% CI 10-13.6) were underweight, and 2.6% (95% CI 1.7-3.5) were wasted. Severe stunting was found in 23.4% (95% CI 21-25.8) of the children; severe underweight in 3.1% (95% CI 2.1-4.1) and severe wasting in 0.6% (95% CI 0.1-1) (Fig. 1).

**Fig. 2.**
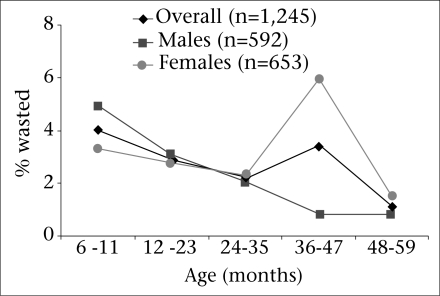
Prevalence of wasting by gender

Severe wasting was most common among children aged 24-35 months, and children aged 24-47 months had the greatest likelihood of being underweight. Moderate wasting was most prevalent (4.1%) among children aged 6-11 months (Table) and was the lowest (1.1%) in children aged 48-59 months. However, there was no statistical association between age and wasting. The proportion (65.7%) of girls wasted was two-fold that of boys (34.4%). These differences were not significant; however, girls aged 36-47 months were significantly (p<0.01) more likely than boys of the same age to have wasting (Fig. 2).

**Fig. 3.**
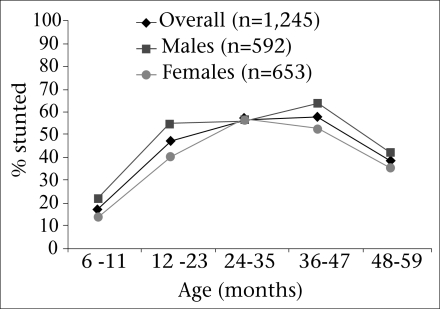
Prevalence of stunting by gender

The prevalence of stunting among children aged 6-59 months was 47%, and the prevalence increased with age through 36-47 months (58%) (Fig. 3). Severe stunting was found in 23.4% of the children. Stunting peaked (56%) in children aged 36-47 months, and compared to other age-groups, stunting was significantly (p<0.01) more likely in children aged 36-47 months. A similar proportion (51.3%) of males were stunted as females (48.7%).

**Table.** Anthropometric measurements in children from Kibera, 2005 and 2008

Anthropometric measurements	Kibera2008					Kibera2008				
	No.	% <-2SD	95%CI	%<-3SD	95%CI	No.	%<-2SD	95%CI	%<-3SD	95%CI
Height/length-for-age—stunting										
Age (months)										
6-11	380	23.4	19-27.8	8.4	5.5-11.3	122	17.2	10.1-24.3	8.2	2.9-13.5
12-23	653	42.6	38.7-46.4	18.1	15-21.1	272	47.1	40.9-53.2	19.9	14.9-24.8
24-35	588	54.6	50.5-58.7	25	21.4-28.6	320	56.3	50.7-61.8	25.9	21-30.9
36-47	543	46.4	42.1-50.7	16.4	13.2-19.6	264	58	51.8-64.1	29.9	24.2-35.6
48-60	592	33.1	29.2-37	9.3	6.9-11.7	267	38.6	32.6-44.6	24.3	19-29.7
Gender										
Male	1,358	41.1	38.6-43.6	16.5	14.6-18.4	592	50.7	46.6-54.8	25.2	21.6-28.7
Female	1,397	35.5	33.1-37.9	13.4	11.7-15.2	653	43.6	39.8-47.5	21.7	18.5-25
Total	2,756	38.3	36.5-40	14.9	13.7-16.2	1245	47	44.2-49.8	23.4	21-25.8
Weight-for-length—wasting										
Age (months)										
6-11	378	10.8	7.6-14.1	5.8	3.3-8.3	122	4.1	0.2-8	0.8	0-2.8
12-23	655	7.8	5.7-9.9	2.4	1.2-3.7	272	2.9	0.7-5.1	1.5	0-3.1
24-35	589	5.1	3.2-7	1.7	0.6-2.8	320	2.2	0.4-3.9	0.3	0-1.1
36-47	544	5.3	3.4-7.3	1.3	0.2-2.3	264	3.4	1-5.8	0.4	0-1.3
48-60	588	8.3	6-10.7	3.1	1.6-4.5	267	1.1	0-2.6	0	0-0.2
Gender										
Male	1,396	7.5	6.1-8.9	2.9	2-3.8	592	2	0.8-3.2	0.7	0-1.4
Female	1,411	6.7	5.9-7.5	2.3	1.5-3.2	653	3.1	1.7-4.5	0.5	0-1.1
Total	2,807	7.1	6.2-8.1	2.6	2-3.2	1245	2.6	1.7-3.5	0.6	0.1-1
Weight-for-age—underweight										
Age (months)										
6-11	388	17	13.1-20.9	7.5	4.7-10.2	122	9	3.5-14.5	1.6	0-4.3
12-23	670	19.7	16.6-22.8	6.9	4.9-8.9	272	10.7	6.8-14.5	2.9	0.7-5.1
24-35	595	24	20.5-27.6	6.1	4.1-8.1	320	14.4	10.4-18.4	4.7	2.2-7.2
36-47	550	21.8	18.3-25.4	6.4	4.2-8.5	264	13.3	9-17.5	4.5	1.8-7.2
48-60	595	20.5	17.2-23.8	6.6	4.5-8.6	267	9.7	6-13.5	0.7	0-2
Gender										
Male	1,383	21.3	19.3-23.3	6.6	5.4-7.9	592	14.4	11.4-17.3	3.4	1.8-4.9
Female	1,415	20.1	18.1-22.1	6.6	5.4-7.9	653	9.5	7.2-11.8	2.9	1.5-4.3
Total	2,798	20.7	19.3-22.1	6.6	5.8-7.5	1245	11.8	10-13.6	3.1	2.1-4.1

CI=Confidence interval;

SD=Standard deviation

The moderate prevalence of underweight (low weight-for-age) was 11.8%; 3.1% of the children were severely underweight. The prevalence of underweight children was the highest (14.7%) among children aged 24-35 months. This difference was not significant when compared with all other age-groups.

The household morbidity surveillance indicated that a quarter of the children had an illness two weeks before the interviews. The prevalence of the top three disease symptoms reported were: respiratory symptoms (46%), fever (25%), and symptoms of gastroenteritis (18%). There were no associations observed between any of the three malnutrition indices and report of recent illness.

Most (89.3%) caregivers reported that they were experiencing food shortages. Families dealt with food shortages by reducing the amount of food eaten (52%) and eating less-expensive food (39%). We asked the caregivers whether they provide less, more, or same amount of foods or drinks when the child is ill. The large majority (73%) of the children ate three or more meals per day. The findings indicate that there were more children (59.4%) who ate less often when sick than those who fed more often when sick; however, there were no significant differences in the feeding practices during illness and nutritional status.

There were significant differences in the nutritional status of children when comparing these data from 2008 with data from a similar survey conducted in 2005 (CDC. Unpublished data) within the same surveillance area and villages in Kibera. Stunting was significantly higher (p<0.01) in 2008 than in 2005 while wasting was significantly higher (p<0.01) in 2005 than in 2008 (Table).

## DISCUSSION

Given the food insecurity during the 2008 post-election crisis when this survey was done, we had expected evidence of widespread acute malnutrition. However, we found that chronic malnutrition was more prevalent than acute malnutrition, raising concerns about persistent and potentially worsening (since our previous survey in 2005) suboptimal nutritional practices in the area. In 2008, the Kenya Demographic and Health Survey found that 28.5% of children in Nairobi were stunted. However, the nationwide survey tends only to sample in areas enumerated during census determinations, which generally under-samples people living in informal settlements. The high (47%) prevalence of stunting among our survey children from a Nairobi slum highlights the risk of not counting those who live in impoverished settings in assessing the urban health and nutritional needs. When compared with our 2005 survey data, the findings from the 2008 survey suggest that there was an increase in the prevalence of stunting of children living in Kibera slum in 2008. The findings of the study highlight that chronic malnutrition is a consistent problem within this informal urban settlement in Kenya. We suspect that these findings are not unique to Kibera and likely to reflect conditions within other informal settlements in Nairobi and other urban settings in Kenya.

Infants aged 6-11 months had a significantly lower risk of being stunted than children in older age-groups. In both study population and 2005 population, it was noted that, after the first year of life, there was a rapid increase in the prevalence of stunting. In the second year of life, with introduction to the family diet, children become more responsible for feeding themselves but often do not have access to adequate amounts of solid food. We found the highest prevalence of stunting in children aged 36-47 months, which was a year older to what we found in the 2005 survey. Our findings are similar to those from a study in India in which stunting was most commonly found in the age-group of 36-47 months, yet was the lowest in children aged 48-59 months. This finding could be attributed to poor weaning and complementary feeding practices, which contribute to inadequate energy and protein intake (13). However, we did not collect data to determine the impact of feeding practices on nutritional status.

We found a higher prevalence of stunting among boys than among girls. This is consistent with the findings of our earlier survey from 2005 (Dooling K *et al.* Unpublished findings). The relationship of chronic malnutrition to age and gender may be linked to the timing and type of complimentary foods introduced in infants’ and toddlers’ diets. These findings are similar to a study of 16 demographic and health surveys in sub-Saharan Africa that revealed that, in 10 countries in sub-Saharan Africa, under-five male children are more likely to become stunted than their female counterparts (14). One report suggested that boys were more influenced by environmental stress than girls (15). If so, boys may be more likely to display impact of chronic undernutrition, especially in environments like informal settlements where various other stresses are at play, like repeated infections and exposure to toxins and air pollutants.

Wasting is usually due to recent illness and/or insufficient dietary intake caused by food shortages, feeding practices, or other events. Provided there is no severe food shortage, the prevalence of wasting is below 5% in most impoverished settings in developing countries, consistent with the findings of this survey.

Undernutrition and childhood morbidity have a synergistic relationship. The interrelationship of the two is in such a way that illness can suppress appetite precipitating undernutrition of a child while, on the other hand, nutritional deficiencies increase the susceptibility of the child to infectious diseases (16). In our study, children reported to be fed less often when sick were more likely to be malnourished than their counterparts who were fed more often when sick. Results of a study in Ghana of infants aged 6-12 months also showed that children who were fed more frequently during illnesses were better-off than those fed less frequently (17).

While we did not find evidence of widespread wasting during the chaos associated with the post-election period, we noted disturbing evidence of a significantly-greater risk for wasting among girls during the third year of their life. Hypothetically, this could be explained by their preference for feeding the male child in times of food scarcity. More data would be needed to determine whether this finding was consistent in other locations and, if so, whether the disparity represents a gender-based differential feeding practices during a period of limited availability of foods.

### Limitations

One significant limitation of our study was that many residents of Kibera left the area during the chaos. The number of children surveyed was also less than half the number surveyed in 2005. Thus, the survey population may have represented a biased sample, possibly weighted towards families who were less acutely affected than families forced to flee away from the area. If so, this would have substantially underestimated the prevalence of acute malnutrition during the crisis.

### Conclusions

Malnutrition among under-five children in the Kibera slums appears to be a sustained crisis instead of an acute, self-limited problem linked to the post-election violence. Stunting is the predominant nutritional problem, and the elevated prevalence in older children indicates failure in growth and development during the first two years of life. The evidence con­tributes to the growing scientific con­sensus that tackling childhood stunting is a high priority and that organizations and governments focusing on preventing malnutrition use integrated approach to include the urban poor. Nutritional supplementation and child health programmes, which are currently focused on impoverished rural areas, should not exclude informal settlements.

## ACKNOWLEDGEMENTS

The authors acknowledge James Ndirangu who analyzed the baseline nutrition data. They also are grateful for the work of the field supervisors—Kennedy Odero and George Okoth, the field workers who collected the data, and the respondents of the study area for their cooperation.
